# An ultra-compact polarization-insensitive slot-strip mode converter

**DOI:** 10.1007/s12200-022-00008-5

**Published:** 2022-04-02

**Authors:** Zihan Tao, Bo Wang, Bowen Bai, Ruixuan Chen, Haowen Shu, Xuguang Zhang, Xingjun Wang

**Affiliations:** 1 State Key Laboratory of Advanced Optical Communication Systems and Networks, School of Electronics, Peking University, Beijing, 100871 China; 2 Frontier Science Center for Nano-Optoelectronics, Peking University, Beijing, 100871 China; 3 Peking University Yangtze Delta Institute of Optoelectronics, Nantong, 226010 China; 4 Peng Cheng Laboratory, Shenzhen, 518055 China

**Keywords:** Silicon photonics, Slot-strip convertor, Multimode interference (MMI), Polarization-insensitive

## Abstract

Integrated waveguides with slot structures have attracted increasing attention due to their advantages of tight mode confinement and strong light-matter interaction. Although extensively studied, the issue of mode mismatch with other strip waveguide-based optical devices is a huge challenge that prevents integrated waveguides from being widely utilized in large-scale photonic-based circuits. In this paper, we demonstrate an ultra-compact low-loss slot-strip converter with polarization insensitivity based on the multimode interference (MMI) effect. Sleek sinusoidal profiles are adopted to allow for smooth connection between the slot and strip waveguide, resulting reflection reduction. By manipulating the MMI effect with structure optimization, the self-imaging positions of the TE_0_ and TM_0_ modes are aligned with minimized footprint, leading to low-loss transmission for both polarizations. The measurement results show that high coupling efficiencies of − 0.40 and − 0.64 dB are achieved for TE_0_ and TM_0_ polarizations, respectively. The device has dimensions as small as 1.1 μm × 1.2 μm and composed of factory-available structures. The above characteristics of our proposed compact slot-strip converter makes it a promising device for future deployment in multi-functional integrated photonics systems.

**Graphic Abstract:**

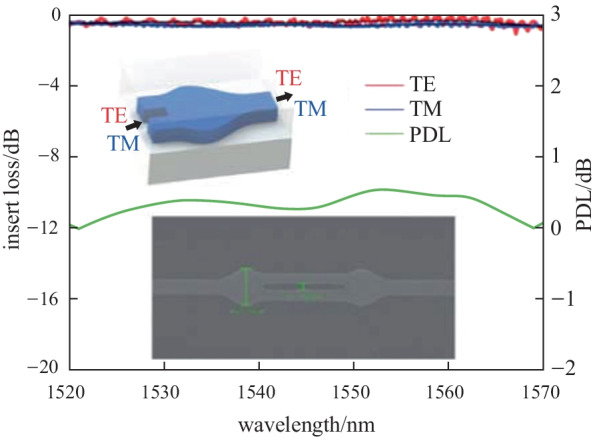

## Introduction

Silicon photonics is emerging as an extensively universal platform due to their integration of various low cost, high-density photonic devices and the maturity of complementary metal-oxide semiconductor (CMOS) manufacturing technology [1–4]. Silicon-based slot waveguides are constructed with a narrow low-index slot (SiO_2_ or air) embedded between two high-index silicon waveguides. Owing to their unique optical field distributions [5–7], slot waveguides are irreplaceable in many applications, such as optical sensors [6], high speed modulators [8, 9], polarization controlling devices [10–12], and nonlinear optical devices [13]. However, the propagation loss of slot waveguides, which is almost one order of magnitude larger than that of strip waveguides [14, 15], poses serious challenges for realizing low loss photonic circuits based solely on slot waveguides. Thus, a compact slot-strip converter is necessary to effectively connect the slot waveguide-based functional device with other photonic components. To date, various schemes have been proposed to achieve high coupling efficiency for slot-strip converters. The adiabatic evolution structure [16–18] is widely used while the sharp tips cannot be avoided during gradual mode conversion, which pose great challenges for high-precision fabrication. For example, multimode interference (MMI) schemes [19, 20] produce reflections due to mutation of the waveguide. Moreover, overly narrow slots (which also existed in Ref. [21]) and non-standard silicon thicknesses also make large-scale production extremely difficult, not to mention the advanced fabrication process. Furthermore, the compactness of most schemes is insufficient. Despite its numerous challenges, polarization multiplexing technology is still an effective approach to enhance the capacity of on-chip optical interconnects [22], making polarization-insensitive mode converters [20] paramount for applications that involve multiple modes [12, 23].

In this paper, a polarization-insensitive slot-strip converter is proposed and demonstrated based on novel sinusoidal-profile MMI, which not only decreases the optical mode field mismatching but also has an ultra-compact device size. The insertion losses for TE_0_ and TM_0_ polarizations are measured as low as 0.40 and 0.64 dB, respectively. The polarization-dependent loss is 0.24 dB, indicating that the converter can handle a variety of applications where both TE_0_ and TM_0_ polarizations are needed. Theories and preliminary simulation results have been roughly investigated in Ref. [24], while this paper will present more detailed principles, simulation, and experimental results.

## Design and principles

**Fig. 1:**
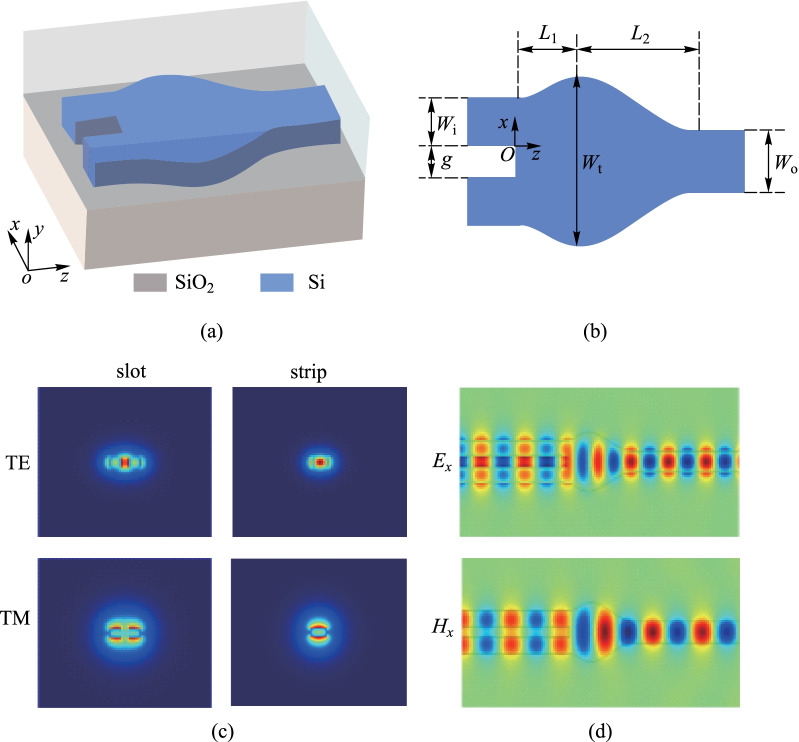
**a** 3D schematic of the device. **b** 2D schematic of the device with structural parameters. **c** 2D optical mode distribution on cross section of slot and strip waveguides. **d** Optical field (*E*_*x*_ or *H*_*x*_) evolution with the input of slot waveguide and output of strip waveguide for both TE_0_ and TM_0_ respectively

As illustrated in Figs. 1(a) and (b), the proposed converter is based on a silicon-on-insulator (SOI) platform, with a 220 nm top Si and 2 μm SiO_2_ cladding. The width (*W*_i_) of the slot waveguide is set at 300 nm for specific needs and the gap (*g*) is fixed at 200 nm, which is compatible with standard 180 nm CMOS processes. The output strip waveguide width (*W*_o_) is 400 nm for single mode operation. The core region, where the MMI effect and optical mode evolution take place from slot mode to strip mode, is shown in Fig. 1(d). In the calculation, {*E*_*tv*_, *H*_*tv*_} represent the transverse optical fields of the slot waveguide, while that of the eigenmodes in the MMI region is denoted as {*E*_*tm*_, *H*_*tm*_}. Based on the coupled mode theory, the mode overlap ratio (Γ) between the two structures can be described as

1 $$\Gamma { = }\frac{{\left| {\frac{1}{4}\iint {[E_{tm} \times H_{t\nu }^{*} + E_{t\nu }^{*} \times H_{tm} ]_{z} {\text{d}}x{\text{d}}y}} \right|^{2} }}{{\frac{1}{4}\iint {[E_{tv} \times H_{tv}^{*} + E_{tv}^{*} \times H_{tv} ]_{z} {\text{d}}x{\text{d}}y \cdot \frac{1}{4}\iint {[E_{tm} \times H_{tm}^{*} + E_{tm}^{*} \times H_{tm} ]_{z} {\text{d}}x{\text{d}}y}}}}.$$

According to Eq. (1), the TE_0_ of slot mode can be expanded into TE_0_ and TE_2_ of strip mode in the MMI region and the self-imaging points would be periodically restructured. For the 800 nm width strip waveguide, the proportion of TE_0_ and TE_2_ is 89.7% and 7.6%, respectively, when the slot mode is injected. The MMI process differs from the previous MMI principle in that the proposed structure is spatially modulated by the profile. Thus, the propagation constant of each mode is variable and the position and proportion of different onefold image modes are different with each change of profile. Therefore, by appropriately designing the shape of the sinusoidal-MMI region, the desired mode distribution can be obtained at the output port.

The core region is composed of two different sinusoids with longitudinal lengths of *L*_1_ and *L*_2_. The junction of these two arcs determines the maximum width (*W*_t_). The function of the profile is

$$x = \frac{{W_{{\text{t}}} - 2W_{{\text{i}}} - g}}{4}\sin \left( {\frac{{\uppi }}{{L_{1} }}z - \frac{{\uppi }}{2}} \right) + C_{0} ,\;z \in (0,L_{1} ],$$

and

$$x = \frac{{W_{{\text{t}}} - W_{{\text{o}}} }}{4}\sin \left( {\frac{{\uppi }}{{L_{2} }}z + \frac{{\uppi }}{2}} \right) + C_{1} ,\;z \in (L_{1} ,L_{2} ].$$

The first order derivative of the sinusoidal arcs is zero at points –π/2 and π/2, which forms a smooth mode transition at the butt-joint area between the slot waveguide and MMI region for reflection reduction. To demonstrate this theory, a conventional structure in which a slot waveguide directly couples to a regular rectangle waveguide (1.2 μm width) as the MMI region was simulated. In comparison, the proposed structure can theoretically achieve a 30% reflection reduction. Other core region profiles in reality demonstrate geometric similarities when the first order derivative is zero. The differences between these structures may not be as apparent following production. Since high performance have been obtained with a sinusoidal function, this simple and easily available profile function was adopted. While previous structures require a tapered structure after the MMI/directly coupling region for single mode operation, our proposed structure does not require the additional tapered region to function, allowing for further size reduction. This is because our design combines the functions of both regions, so that the optical field can be manipulated inside the sinusoidal-MMI and the mode profile of the MMI onefold image highly overlaps with that in the 400 nm single-mode waveguide.

**Fig. 2:**
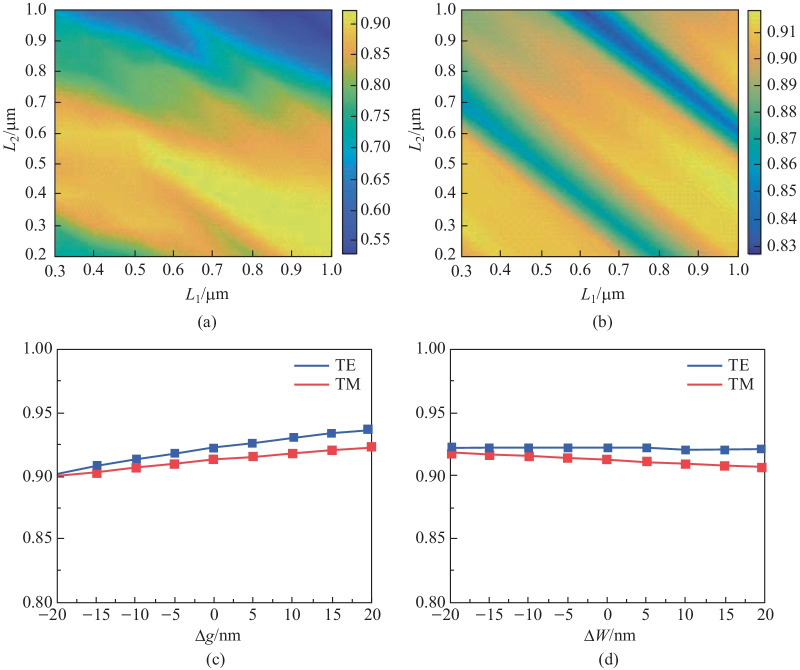
Simulation results of **a** TE_0_ and **b** TM_0_ normalized transmission with different lengths of *L*_1_ and *L*_2_ under the width *W*_t_ of 1.1 μm. **c** and **d** show the simulation results of fabrication tolerance

The ideal way to achieve polarization-insensitive mode conversion is to calculate the self-imaging positions of TE_0_ and TM_0_, where polarization constructs the onefold images. However, this may result in a larger device size since the coincident point may be located further from the starting position. In our design, the first onefold image for both TE_0_ and TM_0_ is the optimal solution because the relatively small distance between the two points can still guarantee low-loss transmission of both polarizations. Optimization of the core region structure was achieved through parameter traversing of {*W*_t_, *L*_1_, *L*_2_} around the first onefold image as shown in Figs. 2(a) and (b), where the figure of merit (FOM) is defined as

2 $${\text{FOM}} = T_{{{\text{TE}}}} \times T_{{{\text{TM}}}} ,$$

where $T_{{{\text{TE}}}}$ ($T_{{{\text{TM}}}}$) represent the normalized transmission of TE_0_ (TM_0_). A high FOM indicates high transmission for both TE_0_ and TM_0_ polarizations. Finally, dimensions of *W*_t_ = 1.1 μm, *L*_1_ = 0.4 μm, and *L*_2_ = 0.8 μm were selected for low-loss polarization-insensitive slot-strip conversion. All 3D simulations presented in this paper were carried out using finite element method (FEM). Figure 2(c) and (d) show the fabrication tolerance analysis. The waveguide width variation, Δ*W* (including the variation of *W*_i_, *W*_t_, and *W*_o_), and the slot gap variation, Δ*g*, are significant parameters when determining the fabrication tolerance. The simulation results indicate that the conversion efficiency decline are less than 0.3% for TE_0_ mode, 0.7% for TM_0_ mode with the variation of *g* within ± 20 nm, and 2.1% for TE_0_, 1.4% for TM_0_ with the variation of *W* within ± 20 nm.

## Fabrication and experimental measurements

**Fig. 3:**
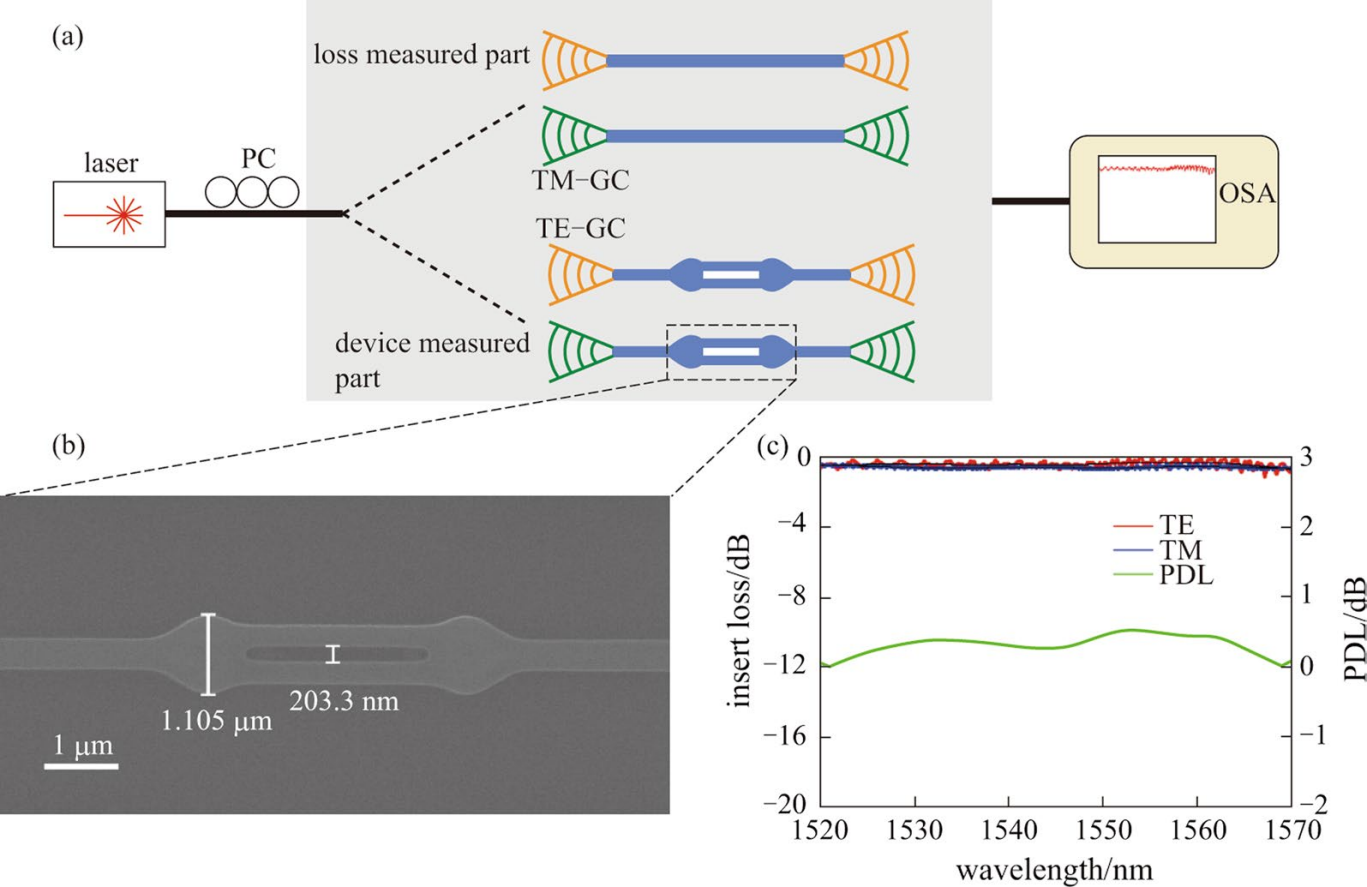
**a** Schematic of the device measurement. PC, polarization control; TM-GC, TM polarization sensitive grating coupler; TE-GC, TE polarization sensitive grating coupler; OSA, optical spectrum analyzer. **b** Top-view SEM picture of fabricated converters. **c** Measured transmission of TE_0_ mode (red line) and TM_0_ mode (blue line); the black line are trend lines by robust locally weight regression method. The green line represents the polarization dependent loss (PDL)

The proposed converter was fabricated in the United Microelectronics Center (CUMEC), based on standard 180 nm SiP process (Fig. 3(a)). Figure 3(b) exhibits the scanning electron microscope (SEM) image of the converter. The total length of the proposed converter is 1.2 μm. A polarization-sensitive grating coupler was used to guide the light into the chip, while maintaining high polarization selectivity for either TE_0_ or TM_0_ modes. To characterize device performance, a tunable continuous wave (CW) laser (AQ 2200-136TLS, YOKOGAWA) was used as the source and the polarization was adjusted using a polarization controller (DPC5500, THORLABS). Then, the light was coupled into the chip through the polarization-sensitive grating coupler. An optical spectrum analyzer (AQ 6370C, YOKOGAWA) was used to measure the output spectrum coupled from the output grating coupler. Figure 3(c) shows the measured transmission for both TE_0_ and TM_0_ modes, which are extracted by normalizing the measured spectra with a 400 nm width waveguide to exclude the loss of grating coupling.

The normalized spectra still show a small amount of reflection from the butt-joint since the ripple of the spectrum especially for TE_0_, is observed as a result of Fabry–Perot resonance. The robust locally weighted regression method has been implemented [25] to suppress the noise (shown as solid black lines in Fig. 3(c)). The insertion loss (IL), defined as ${\text{IL}}_{{{\text{TE}}_{0} ,{\text{TM}}_{0} }} = 10\log_{10} \left( {P_{{{\text{TE}}_{0} ,{\text{TM}}_{0} }} /P_{{{\text{in}}}} } \right)$, is used to characterize the transmission loss. At approximately the 1550 nm wavelength, the IL is − 0.40 and − 0.64 dB (single-ended loss) for TE_0_ and TM_0_, respectively, indicating that the proposed converter is capable of low loss transmission for both polarizations. The polarization-dependent loss (PDL), defined as ${\text{PDL}} = \left| {{\text{IL}}_{{{\text{TE}}}} - {\text{IL}}_{{{\text{TM}}}} } \right|$, is used to characterize the polarization insensitivity of the converter. The PDL of the converter is about 0.24 dB, meaning it can support most polarization-insensitive applications. Compared to other studies, the converter proposed in this paper is the most compact in size, though the IL may be slightly lower than others in Refs. [19, 20]. However, according to the coupled mode theory, a wider slot can exacerbate mode mismatch, as illustrated in Ref. [21]. With that said, we still need to overcome many challenges in order to eliminate mode mismatch for a 200 nm slot, the widest of all previous works.

## Conclusion

In conclusion, this paper numerically and experimentally demonstrates an ultra-compact polarization-insensitive slot-strip converter that takes advantage of low-loss transmission of the MMI effect. Due to the characteristic shape of the sinusoidal arcs, the converter can create a smooth connection between the slot and single-mode strip waveguide, with only slight reflections. The proposed device is easily manufactured, with a compact dimensions of 1.1 μm × 1.2 μm. The IL for both TE_0_ and TM_0_ polarizations is lower than 0.65 dB and the PDL is about 0.24 dB at around 1550 nm. These characteristics are advantageous for various applications that require both TE_0_ and TM_0_ polarizations, such as optical sensing, high speed modulation, and polarization controlling devices.
