# Correction to: An ultra-compact polarization-insensitive slot-strip mode converter

**DOI:** 10.1007/s12200-022-00031-6

**Published:** 2022-08-10

**Authors:** Zihan Tao, Bo Wang, Bowen Bai, Ruixuan Chen, Haowen Shu, Xuguang Zhang, Xingjun Wang

**Affiliations:** 1 State Key Laboratory of Advanced Optical Communication Systems and Networks, School of Electronics, Peking University, Beijing, 100871 China; 2 Frontier Science Center for Nano-Optoelectronics, Peking University, Beijing, 100871 China; 3 Peking University Yangtze Delta Institute of Optoelectronics, Nantong, 226010 China; 4 Peng Cheng Laboratory, Shenzhen, 518055 China

## Correction to: Frontiers of Optoelectronics (2022) 15:5 10.1007/s12200-022-00008-5

Following publication of the original article [1], the authors identified an error in Fig. 3b. The correct Fig. 3 is in this correction, and the original article has been corrected.

**Fig. 3:**
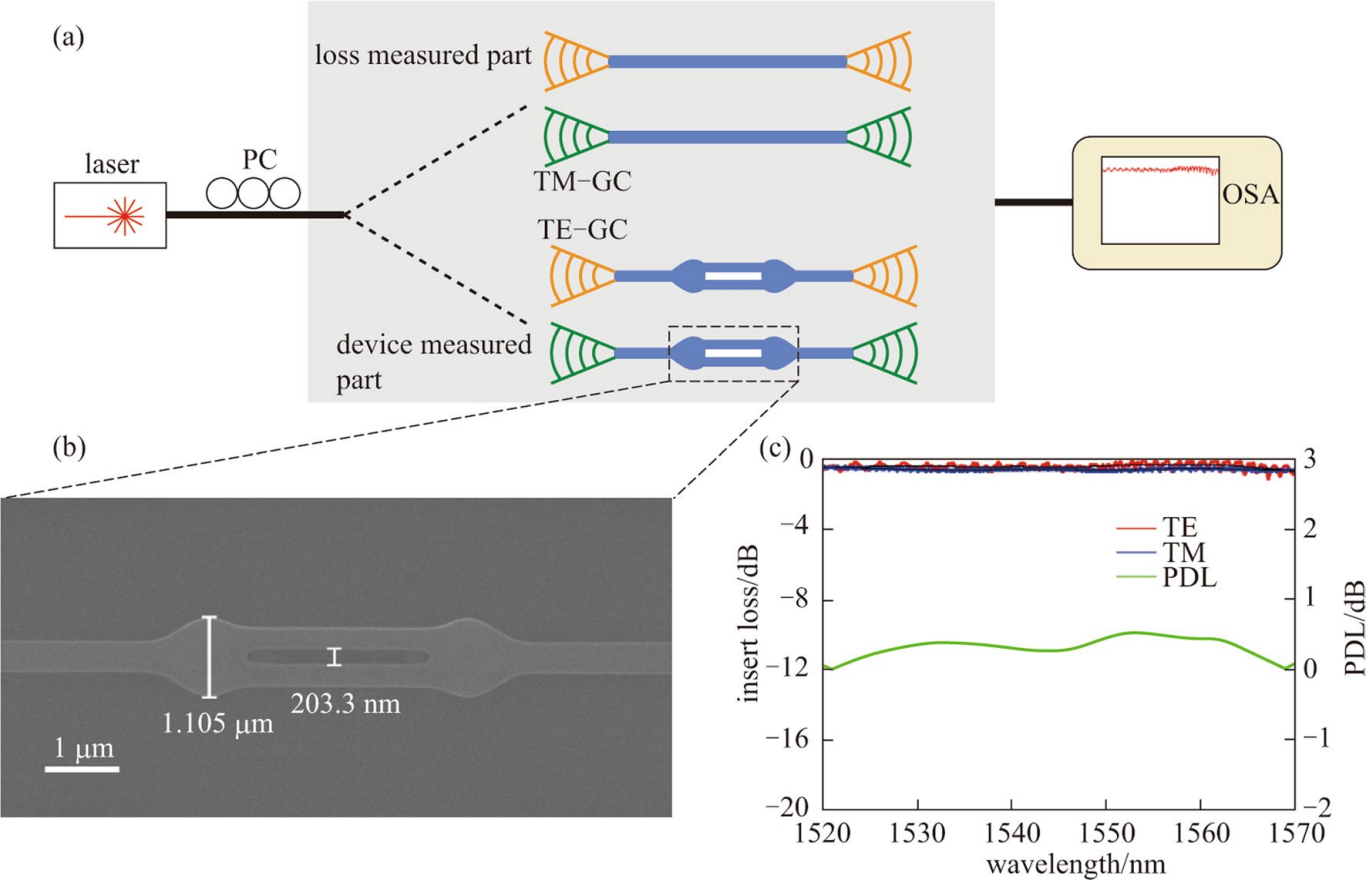
**a** Schematic of the device measurement. PC, polarization control; TM-GC, TM polarization sensitive grating coupler; TE-GC, TE polarization sensitive grating coupler; OSA, optical spectrum analyzer. **b** Top-view SEM picture of fabricated converters. **c** Measured transmission of TE0 mode (red line) and TM0 mode (blue line); the black line are trend lines by robust locally weight regression method. The green line represents the polarization dependent loss (PDL)
